# Ghrelin Attenuates the Osteoblastic Differentiation of Vascular Smooth Muscle Cells through the ERK Pathway

**DOI:** 10.1371/journal.pone.0033126

**Published:** 2012-04-13

**Authors:** Qiu-Hua Liang, Yi Jiang, Xiao Zhu, Rong-Rong Cui, Guan-Ying Liu, Yuan Liu, Shan-Shan Wu, Xiao-Bo Liao, Hui Xie, Hou-De Zhou, Xian-Ping Wu, Ling-Qing Yuan, Er-Yuan Liao

**Affiliations:** 1 Institute of Metabolism and Endocrinology, the Second Xiang-Ya Hospital, Central South University, Changsha, People's Republic of China; 2 Department of Pathology, the Second Xiang-Ya Hospital, Central South University, Changsha, People's Republic of China; 3 Department of Pediatrics, the Second Xiang-Ya Hospital, Central South University, Changsha, People's Republic of China; 4 Department of Cardiothoracic Surgery, the Second Xiang-Ya Hospital, Central South University, Changsha, People's Republic of China; Universitat de Lleida – IRBLLEIDA, Spain

## Abstract

Vascular calcification results from osteoblastic differentiation of vascular smooth muscle cells (VSMCs) and is a major risk factor for cardiovascular events. Ghrelin is a newly discovered bioactive peptide that acts as a natural endogenous ligand of the growth hormone secretagog receptor (GHSR). Several studies have identified the protective effects of ghrelin on the cardiovascular system, however research on the effects and mechanisms of ghrelin on vascular calcification is still quite rare. In this study, we determined the effect of ghrelin on osteoblastic differentiation of VSMCs and investigated the mechanism involved using the two universally accepted calcifying models of calcifying vascular smooth muscle cells (CVSMCs) and beta-glycerophosphate (beta-GP)-induced VSMCs. Our data demonstrated that ghrelin inhibits osteoblastic differentiation and mineralization of VSMCs due to decreased alkaline phosphatase (ALP) activity, Runx2 expression, bone morphogenetic protein-2 (BMP-2) expression and calcium content. Further study demonstrated that ghrelin exerted this suppression effect via an extracellular signal-related kinase (ERK)-dependent pathway and that the suppression effect of ghrelin was time dependent and dose dependent. Furthermore, inhibition of the growth hormone secretagog receptor (GHSR), the ghrelin receptor, by siRNA significantly reversed the activation of ERK by ghrelin. In conclusion, our study suggests that ghrelin may inhibit osteoblastic differentiation of VSMCs through the GHSR/ERK pathway.

## Introduction

Vascular calcification, previously viewed as a passive consequence of aging [1], is often found in patients with atherosclerosis, diabetes, renal failure and heart failure. It is also associated with many clinical complications such as myocardial infarction, impaired vascular tone, angioplasty dissection and poor surgical outcome [2], [3]. There is now a growing awareness that vascular calcification is an active biologically regulated phenomenon that is similar to bone formation [4], [5]. This process includes the expression of several osteoblast phenotype genes such as alkaline phosphatase (*ALP*), core binding factor α1 (*cbfα1*, *Runx2*), osteocalcin and osteopontin, and requires the presence of the bone mineral hydroxyl apatite and matrix vesicles [6]. Previous studies have demonstrated the significant role of vascular smooth muscle cells (VSMCs) in the active regulation of vascular calcification by their acquisition of the phenotype of osteoblast-like cells [7], [8]. However, the mechanisms of this active process have not been fully explained.

Ghrelin is a 28 amino acid acyl peptide that is esterified with octanoic acid on Ser 3 and that belongs to the brain–gut peptide family; it has been isolated from both human and rat stomach and was first reported by Kojima in 1999 [9]. Subsequent studies have identified that the stomach is the major source of circulating ghrelin, but small amounts of ghrelin are also produced by other organs such as the heart, lung or kidney [10], [11]. Ghrelin has multiple important physiological effects such as promotion of growth hormone (GH) release from the pituitary, increase of food intake, and regulation of energy homeostasis. In addition, recent studies have demonstrated that ghrelin participates in many other physiological processes that include circulation, inflammation, glucose metabolism, cell proliferation, differentiation and apoptosis [12], [13]. Furthermore, it has been shown that the endocrine activities of ghrelin are mediated by the GH secretagog (GHS) receptor (GHS-R). GHS-R is a G-protein-coupled receptor that is mainly expressed in the pituitary gland and in the hypothalamus and that has two subtypes: the fully functional GHSR-1a and the biologically inactive GHSR-1b [14], [15]. As stated earlier, ghrelin is expressed by many organs and tissues especially those of cardiovascular system [16]. In addition, it has been shown to play a role in various cardiovascular activities such as increase in myocardial contractility, decrease in mean arterial pressure, vasodilatation, protection against myocardial infarction-induced heart failure, improvement of ventricular remodeling, as well as attenuation of injury in the heart from ischemia/reperfusion. Recent studies have demonstrated that ghrelin also plays an important role in osteoblast proliferation and differentiation [17], [18]; these results suggest that ghrelin may exert paracrine/autocrine effects in cardiovascular calcification. Recently, Li et al. [19] reported that ghrelin could attenuate vascular calcification through inhibition of osteoblastic differentiation of VSMCs; however the mechanism of this inhibition is still not clear. Calcifying VSMCs (CVSMCs) are a specific subpopulation of VSMCs that can spontaneously express the osteoblast phenotype gene and form calcification nodules [20]. Additionally, normal primary VSMCs cultured with beta-glycerophosphate (beta-GP) can be induced into the process of osteoblastic differentiation. In the present study, both of these cell models were used to explore the effect of ghrelin on osteoblastic differentiation of VSMCs. Our data suggest that ghrelin may attenuate vascular calcification through an extracellular signal-related kinase (ERK)-dependent pathway.

## Results

### Ghrelin inhibited the osteoblastic differentiation and mineralization of VSMCs

**Figure 1:**
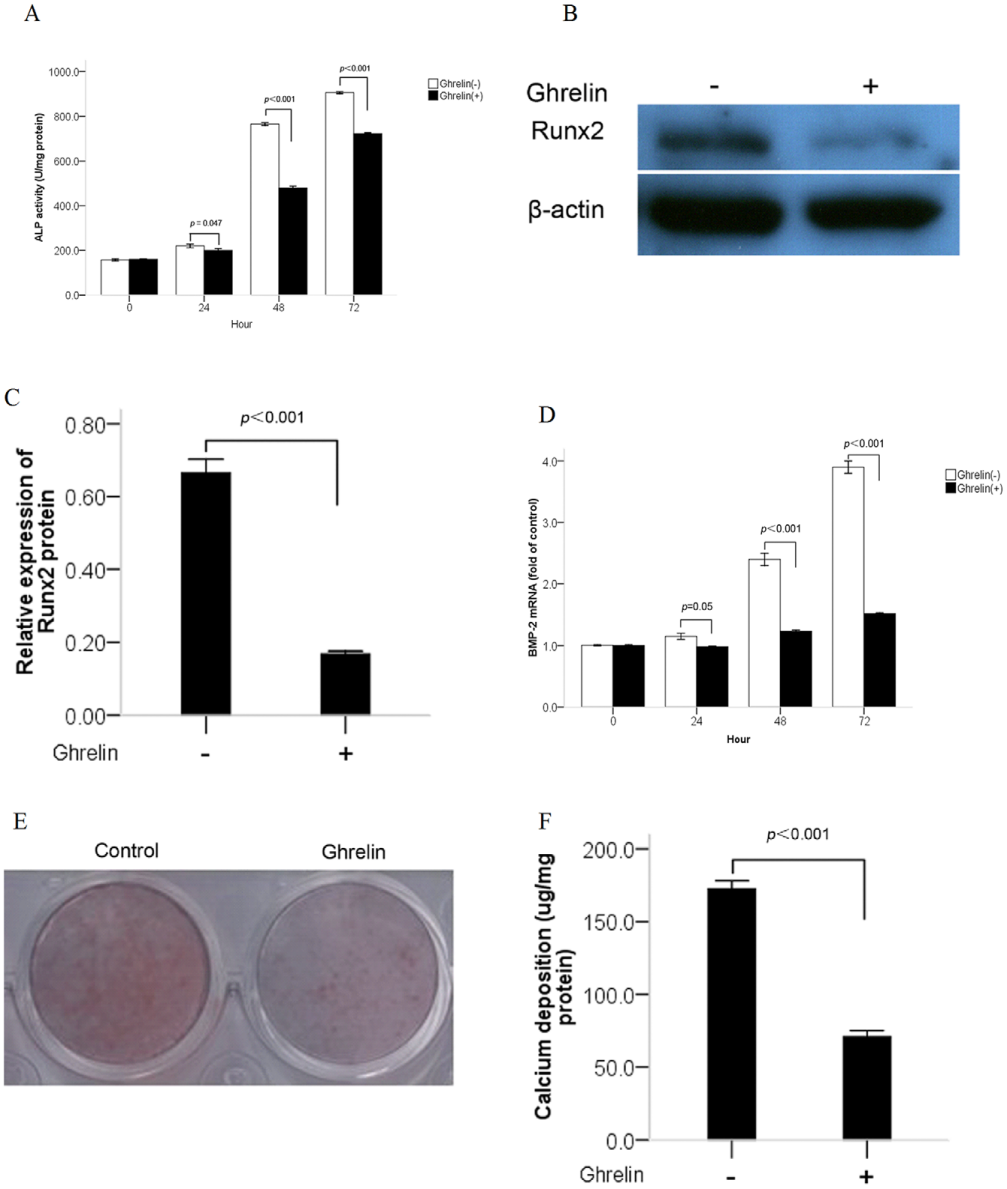
Effects of ghrelin on alkaline phosphatase (ALP) activity, Runx2 protein expression, bone morphogenetic protein-2 (BMP-2) mRNA expression and calcium deposition in calcifying vascular smooth muscle cells (CVSMCs). (A) Effect of ghrelin on ALP activity. The cells were cultured with or without 10−6 mol/L ghrelin for the indicated time periods. ALP activity was measured by an ALP kit, normalized to the cellular protein contents, and presented as mean ±standard deviation (SD) (*n* = 3). (B, C) Effect of ghrelin on Runx2 expression. The cells were cultured for 48 h with or without 10−6 mol/L ghrelin and presented as mean ±SD (*n* = 3). The expression of Runx2 was measured by western blotting. (D) Effect of ghrelin on BMP-2 mRNA expression. The cells were cultured with or without 10−6 mol/L ghrelin for the indicated time periods. BMP-2 mRNA expression was determined by real-time quantitative polymerase chain reaction (qPCR). Results are expressed as fold of control. Bars present mean ±SD (*n* = 3). (E) A representative entire plate view of the Alizarin Red S staining in 24-well plates for control cells and cells treated with ghrelin in 12-day cultures. (F) Effect of ghrelin on calcium deposition. The cells were cultured for 12 days with or without 10−6 mol/L ghrelin and presented as mean ±SD (*n* = 3). The calcium contents of the cell layers were measured by the atomic absorption spectroscopy method.

**Figure 2:**
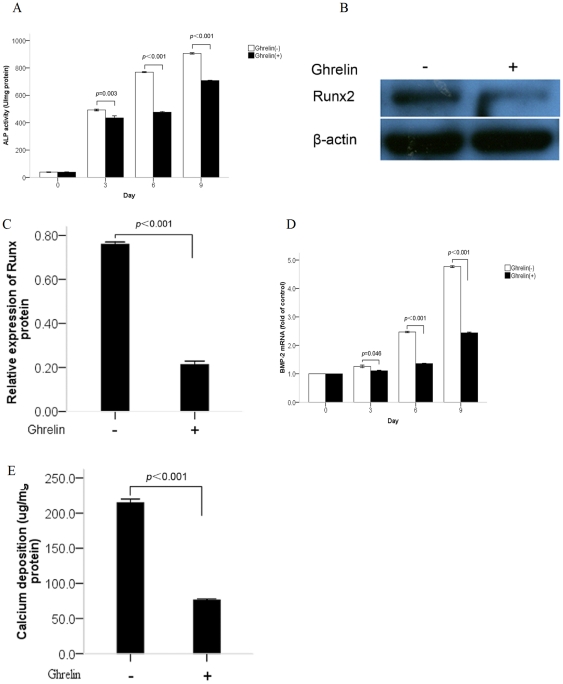
Effects of ghrelin on alkaline phosphatase (ALP) activity, Runx2 protein expression, bone morphogenetic protein-2 (BMP-2) mRNA expression and calcium deposition in vascular smooth muscle cells (VSMCs) cultured with beta-GP. (A) Effect of ghrelin on ALP activity. The cells were cultured with or without 10−6 mol/L ghrelin for the indicated time periods. ALP activity was measured by an ALP kit, normalized to the cellular protein contents, and presented as mean ± standard deviation (SD) (*n* 3). (B, C) Effect of ghrelin on Runx2 expression. The cells were cultured for 6 days with or without 10−6 mol/L ghrelin above and presented as mean ±SD (*n* = 3). The expression of Runx2 was measured by western blot. (D) Effect of ghrelin on BMP-2 mRNA expression. The cells were cultured with or without 10−6 mol/L ghrelin for the indicated time periods. BMP-2 mRNA expression was determined by real-time quantitative polymerase chain reaction (qPCR). Results are expressed as fold of control. Bars represent mean ±standard deviation (SD) (*n* = 3). (E) Effect of ghrelin on calcium deposition. The cells were cultured for 20 days with or without 10−6 mol/L ghrelin and presented as mean ±SD (*n* = 3). The calcium contents of the cell layers were measured by the atomic absorption spectroscopy method.

It is acknowledged that the process of vascular calcification is similar that of bone mineralization, in which both ALP (the phenotypic marker for osteoblastic differentiation) and Runx2 (the transcription factor for osteoblastic differentiation and bone formation) are up-regulated significantly [21]. The bone morphogenic proteins (BMPs) are important regulators of orthotopic bone formation and two members of this group, BMP-2, and -4, are associated frequently with vascular calcification [22]. In this study ALP activity, and expression of BMP-2 and Runx2 were determined to identify the inhibitory effects of ghrelin on osteoblastic differentiation of VSMCs. Our data demonstrated that treatment with ghrelin significantly inhibited ALP activity and that the suppression was time dependent. In CVSMCs, significant inhibition by ghrelin was first observed at 24 h and inhibition peaked at 48 h. Details of the effects of ghrelin on ALP activity in CVSMCs are shown in Figure 1A. In contrast, in VSMCs cultured with beta-GP, significant inhibition by ghrelin was observed on days 3 to 9 and inhibition peaked on day 6. Details of ALP activity in VSMCs cultured with beta-GP are shown in Figure 2A.

The expression of Runx2 was determined by western blotting, which was similar to that found for ALP in CVSMCs and VSMCs cultured with beta-GP. Expression of Runx2 was inhibited significantly after treatment with ghrelin for 48 h for CVSMCs (Figure 1B and 1C) and 6 days for VSMCs (Figure 2B and 2C), respectively.

BMP-2 expression was determined by real-time quantitative polymerase chain reaction (qPCR). Our data showed that BMP-2 mRNA expression in CVSMCs and VSMCs was similar to that for Runx2. Details are shown in Figure 1D and Figure 2D.

Figure 1E shows an entire plate of cells stained with Alizarin Red S. Treatment of CVSMCs with ghrelin in culture for 12 days significantly decreased the level of Alizarin Red S staining. Similarly, the effects of ghrelin on calcium deposition in CVSMCs were determined after incubation for 12 days and the data showed that ghrelin markedly decreased calcium deposition in CVSMCs (Figure 1F). In addition, the inhibitory effects of ghrelin on calcium deposition in VSMCs cultured with beta-GP were also determined after a 20-day incubation (Figure 2E) and the results showed the same trends as those for CVSMCs.

**Figure 3:**
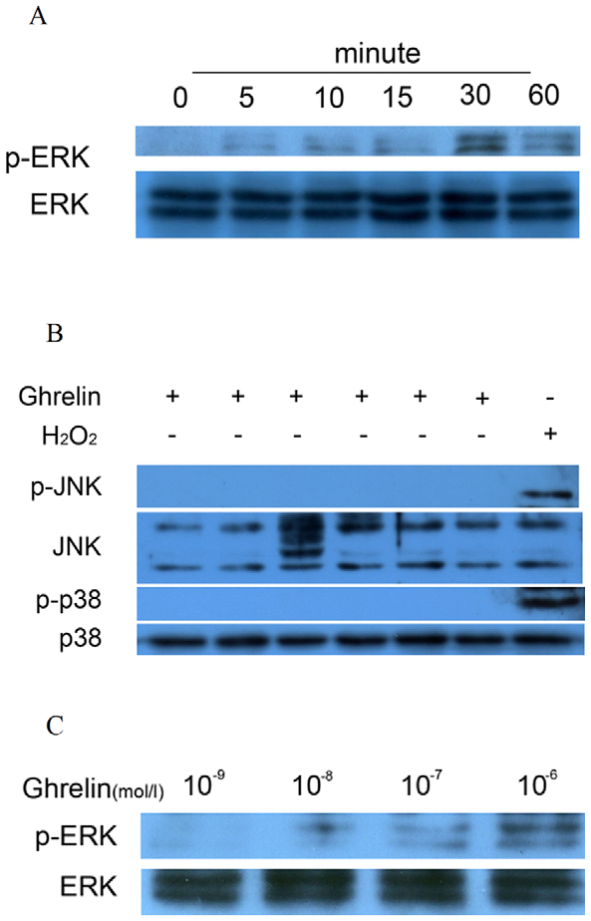
Effects of ghrelin on mitogen-activated protein kinase (MAPK) activation in calcifying vascular smooth muscle cells (CVSMCs). (A, B) Cells were exposed to 10−6 mol/L ghrelin for 0–60 min, or 50 µM hydrogen peroxide (H2O2) for 15 min as a positive control for p38 and JNK activation. Cell lysates were subjected to western blotting and incubated with antibodies against p-ERK, ERK, p-p38, p38, p-JNK, and JNK. The representative results are shown. (C) Cells exposed to 10−9 mol/L, 10^−8^ mol/L, 10^−7^ mol/L, and 10^−6^ mol/L of ghrelin for 30 min. Cell lysates were subjected to western blotting and incubated with antibody against p-ERK and ERK. The representative results are shown.

### Intracellular signaling mechanisms under stimulus of ghrelin

Mitogen-activated protein kinases (MAPKs) play an essential role in the control of cell differentiation and the MAPK signaling pathway is known to be involved in osteoblastic differentiation of VSMCs [4]. To confirm the involvement of MAPK signaling molecules and explore the effects of ghrelin on osteoblastic differentiation, the expression of three MAPK molecules, ERK, c-Jun N-terminal kinases (JNK) and p38 MAPK (p38), were determined in a calcification model and by ghrelin treatment. Our data demonstrated that ERK was phosphorylated significantly by ghrelin, but both JNK and p38 did not respond to ghrelin stimulus but did respond to the positive control hydrogen peroxide (H2O2). Specifically for CVSMCs, ERK activation occurred at 5 min after the start of incubation and peaked at 30 min (Figure 3A and 3B), which suggested that the effects of ghrelin on osteoblastic differentiation of CVSMCs may be mediated by ERK. A range of concentrations of ghrelin (10−9 mol/L, 10^−8^ mol/L, 10^−7^ mol/L, and 10^−6^ mol/L) were added to the CVSMCs culture for 30 min to determine their effect on ERK activation. Our results showed that ghrelin at a concentration that ranged from 10^−8^ mol/L to 10^−6^ mol/L significantly activated ERK and that the activation was dose dependent (Figure 3C), but that a concentration of 10−9 mol/L had no effect on ERK activation.

### Ghrelin attenuated osteoblastic differentiation of CVSMCs through the GHSR/ERK signaling pathway

Silencing of GHSR by siRNA and blockade of ERK phosphorylation by its inhibitor, PD98059, were performed to confirm that the inhibition of calcification of CVSMCs by ghrelin was dependent on phosphorylation of ERK. Expression of both GHSR and ERK were analyzed by western blotting. ALP activity, Runx2 mRNA levels and calcium deposition were also measured.

**Figure 4:**
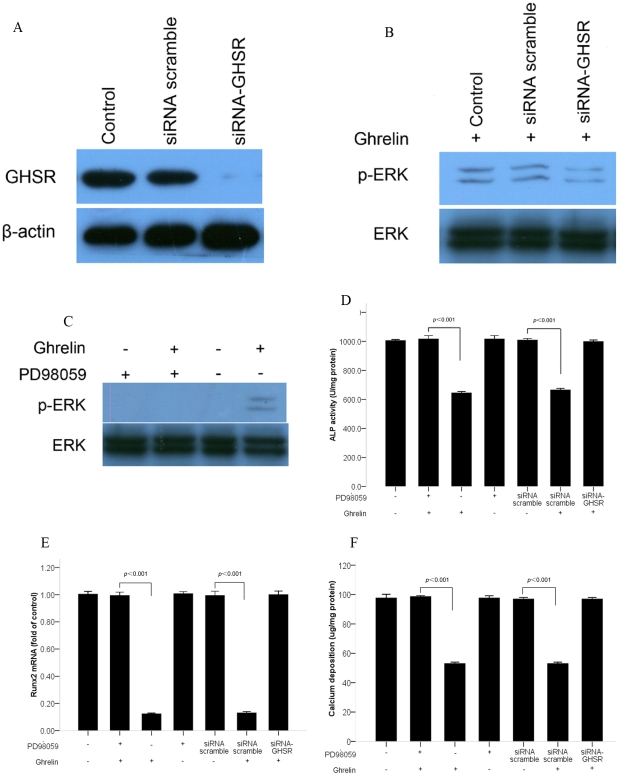
ERK signaling pathway mediated the inhibitory effect of ghrelin on osteoblastic differentiation of calcifying vascular smooth muscle cells (CVSMCs). (A) The expression and silencing of growth hormone secretagog receptor (GHSR) on CVSMCs. Cells were incubated with scramble siRNA or GHSR siRNA for 72 h. Total cellular protein was subjected to western blot analysis using anti-GHSR antibody. The anti-GHSR antibody identified a band at 44 kDa. β-actin was used as the control. (B) The activation of extracellular signal-related kinase (ERK) under the silencing of GHSR. Cells were incubated with scramble siRNA or GHSR siRNA for 72 h prior to treatment with 10−6 mol/L ghrelin for 30 min. Total proteins were subjected to western blotting and incubated with antibody against p-ERK and ERK. The representative results are shown. (C) The activation of ERK under PD98059. Cells were incubated with PD98059 (10 µM) for 2 h prior to treatment with 10−6 mol/L of ghrelin for 30 min. Total proteins were subjected to western blotting and incubated with antibody against p-ERK and ERK. The representative results are shown. (D, E, F) The decreased alkaline phosphatase (ALP) activity, Runx2 mRNA, and calcium deposition mediated by the GHSR/ERK pathway. Cells were incubated with PD98059 (10 µM) for 2 h prior to treatment with 10−6 mol/L of ghrelin. Cells were also treated with the scramble siRNA or GHSR siRNA in the presence of 10−6 mol/L of ghrelin. ALP activity, Rnux2 mRNA, and calcium deposition were measured. The bars represent the mean ±standard deviation (SD) (*n* = 3).

Our data confirmed that expression of the GHSR protein is blocked significantly by siRNA; interestingly, silencing of GHSR also significantly inhibited ERK phosphorylation and abolished the inhibition of ALP activity, Runx2 mRNA production and calcium deposition stimulated by 10^–6^ mol/L ghrelin. ERK inhibitor reversed the decrease of ALP activity, Runx2 mRNA production, and calcium deposition mediated by ghrelin, which indicated that the phosphorylation of ERK was involved in ghrelin signaling. Details are shown in Figure 4.

## Discussion

Vascular calcification, which includes coronary and aortic calcification, is an important risk factor for cardiovascular morbidity and mortality [2]. Clarification of the definite mechanisms for vascular calcification and development of effective therapeutic strategies are both challenging areas of research.

Previous studies by our group have shown that the administration of apelin, taurine, omentin and adiponectin attenuated the calcification of CVSMCs [23]–[25]. At present, two universally accepted cell models are used to study osteoblastic differentiation of VSMCs *in vitro*. In the first model, VSMCs are induced by beta-GP [19], [26], [27]. In the second, around 20–30% VSMCs undergo and form calcified matrix spontaneously *in vitro* without a phosphate donor in the medium, which is called CVSMCs and have been used to investigate osteoblastic differentiation mechanism in our and other's previous studies [22]–[25], [28]. This cell model produces cells that have a more typical osteoblast phenotype, as cells can express ALP, osteocalcin, Runx2, and automatically form calcified matrix nodules. In addition, these cells express osteoblast-related genes (*ALP*, *Runx2*) more quickly than do beta-GP-induced VSMCs as demonstrated in previous studies [28], [29] and in the present work. Therefore, we decided to use a maximum 72 h period for CVSMCs and up to 9 days for beta-GP-induced VSMCs to examine the osteoblastic differentiation of these cells.

In the present study, VSMCs were cultured with ghrelin for different lengths of time and important phenotypic markers of osteoblastic differentiation were detected. A previous study observed that 10−8–10−6 mol/L ghrelin treatment for 10 days decreased the ALP activity of calcified VSMCs in a dose-dependent manner [19]; here we demonstrated that the decrease of ALP activity by ghrelin also may be time dependent. Our data showed that treatment of CVSMCs with 10−6 mol/L of ghrelin inhibited ALP activity from 24 h up to 72 h, the most significant inhibition of ALP activity occurred at 48 h. Furthermore, incubation with 10−6 mol/L of ghrelin for 3 days inhibited the ALP activity of VSMCs induced by beta-GP. It has been shown previously that Runx2 suppresses myocardin-induced smooth muscle cell (SMC) phenotype genes and concomitantly promotes osteogenic conversion of SMCs [30]. Nakahara et al. [31] demonstrated that fibroblast growth factor 2 (FGF-2) induction of osteoblast-marker gene expression was mediated by an increase in DNA-binding activity of Runx2. Recently, Byon et al. [29] have reported that Runx2 promoted the osteoblastic differentiation of macrophages by up-regulating the expression of receptor activator of nuclear factor kappa B ligand (RANKL), which mediates the crosstalk between CVSMCs and migration and differentiation of macrophages into osteoclast-like cells in the atherosclerotic lesions. Taken together, these results support the proposal that Runx2 may be a key regulatory factor in vascular calcification. It has been reported that treatment with 10−7 mol/L ghrelin for 7 days significantly suppressed the expression of Runx2 in preosteoblast C3H10T1/2 cells, while another independent experiment showed that ghrelin did not affect the expression of Runx2 in the differentiation of rat osteoblasts [18], [32]. Our results demonstrated that the expression of Runx2, an important transcription factor for osteoblastic differentiation, and calcium deposition are both down-regulated significantly by ghrelin. The effects of ghrelin may be tissue or cell specific. BMP-2 is expressed in VSMCs and can induce the expression of Runx2 in the process of osteoblastic differentiation of VSMCs [22]. Our quantitative real-time PCR data showed that BMP-2 mRNA expression is significantly down-regulated by ghrelin in CVSMCs and VSMCs induced by beta-GP. Furthermore, the effect was time dependent. Taking together the previous studies and the present results, we think that ghrelin may affect osteoblastic differentiation of VSMCs in both a dose- and time-dependent manner. It has been demonstrated by our group that treatment with apelin for 48 h decreased the ALP activity of CVSMCs significantly and inhibited osteoblastic differentiation of CVSMCs, accompanied by suppression of Runx2 expression [23]. Our present experiment demonstrated that ghrelin, which might act in a similar manner to apelin, inhibited osteoblastic differentiation of VSMCs.

To gain further insight into the mechanism by which ghrelin inhibits osteoblastic differentiation of CVSMCs, we evaluated signaling pathway events. The MAPKs, including ERK, JNK and p38, are serine/threonine kinases that are involved mainly in the activation of nuclear transcription factors that control cell proliferation, differentiation and apoptosis [23], [33]. Several previous studies have shown that ERK is involved in osteoblastic differentiation and mineralization of VSMCs, but this involvement is still controversial. Radcliff et al. [28] suggested that IGF-I inhibited osteoblastic differentiation and vascular-cell mineralization via both the ERK and PI3K pathways. Liu et al. [20] reported that selenite suppressed H_2_O_2_-enhanced osteoblastic differentiation and calcification of VSMCs through the ERK pathway. Shi et al. [26] found that LaCl_3_ suppressed the beta-GP-induced osteoblastic differentiation and calcification of rat VSMCs by the activation of both ERK and JNK MAPK pathways. However, Ding et al. [27] demonstrated that fibronectin enhanced osteoblastic differentiation of VSMCs via the ERK signaling pathway. Our present study suggested that ghrelin inhibited osteoblastic differentiation and mineralization of CVSMCs via the ERK signaling pathway, a finding that is in accordance with Radcliff's, Liu's, and Shi's work and our previous studies [4], [5], [23]. However, we found no activation of the other kinases JNK and p38 in this experiment. Our data showed that 10−9 mol/L of ghrelin had no stimulus effect on ERK activation, but that a dose of 10−8 to 10−6 mol/L of ghrelin stimulated the expression of p-ERK, and therefore that the stimulus is dose dependent. Furthermore, both silencing of GHSR and blockade of ERK suppressed the inhibition of ghrelin on ALP activity, Runx2 mRNA expression and calcium content. Overall, our study suggested that the ghrelin/GHSR signaling pathway may attenuate osteoblastic differentiation of CVSMCs through an ERK-dependent pathway.

In summary, our study identified the effects of ghrelin on osteoblastic differentiation of VSMCs and demonstrated that ghrelin exerted this suppression by promotion of the phosphorylation of ERK. Our findings suggested that ghrelin may be a prospective target for vascular calcification therapy. However, further study is necessary before any clinical application is considered.

## Materials and Methods

### Reagents

Synthetic mouse ghrelin peptide (Gly-Ser-Ser (n-octanoyl)-Phe-Leu-Ser-Pro-Glu -His-Gln-Lys-Ala-Gln-Gln-Arg-Lys-Glu-Ser-Lys-Lys-Pro-Pro-Ala-Lys-Leu-Gln-Pro-Arg) was purchased from the Chinese Peptide Company (Hangzhou, China). Anti-mouse Runx2 antibody, anti-mouse monoclonal IgG peroxidase conjugate antibody, anti-β-actin polyclonal antibody, anti-ERK, p-ERK, p-p38, p38, JNK, p-JNK antibodies, and anti-mouse GHSR antibody were purchased from Santa Cruz Biotechnology Inc (Waltham, MA, USA). ERK inhibitor PD98059 was purchased from Calbiochem Corp. (San Diego, CA, USA). The ALP assay kit was purchased from Nanjing Jiancheng Bioengineering Institute (Nanjing, China). Mouse GHSR siRNA was purchased from GenePharma Company (Shanghai, China).

### Cell culture and *in vitro* calcification

Mouse VSMCs were acquired by an explant method as our previous described [23], [25], and after approved by the Ethics Committee of the Second Xiangya Hospital of Central South University, China. Briefly, the tunica media was isolated from the mouse aortas. The tissue was fragmented (1–2 mm3), the aortas were minced and digested in 5 ml of digestion solution (0.125 mg/ml elastase, 0.25 mg/ml soybean trypsin inhibitor, 10 mg/ml collagenase I, 2.0 mg/ml crystallized bovine albumin, and 15 mM HEPES) at 37°C for 45 min. The cellular digests were filtered through sterile 100-mMnylon mesh, centrifuged at 1,000 rpm for 10 min, and washed twice in Dulbecco's Modified Eagle's medium (DMEM) containing 10% FBS (Gibico-BRL Corp., NY, USA) before culture in the same medium. CVSMCs were isolated from cultures in which multicellular nodules spontaneously appeared. From these nodule-forming cultures, cells were cloned by limiting dilution and single cell harvesting. Colonial lines were identified as CVSMCs by their positive staining with monoclonal antibody α-actin and by their ability to express high levels of ALP and form calcified nodules. Immunocytochemical examination showed positive staining in all cells for α-smooth muscle actin [23]. CVSMCs were seeded in DMEM containing 4.5 g/L of glucose, 10% FBS and 10 mM sodium pyruvate and the medium was refreshed every 2 days. VSMCs induced by beta-GP were cultured in above mentioned medium including 10 mM beta-GP. We used 10−9 mol/L to 10−6 mol/L of ghrelin to incubate VSMCs in the following experiment. The dose of ghrelin was selected based on the previous research [19]. After incubation, the following measurements were performed.

### Analysis of ALP activity

Cells were cultured in the absence or presence of 10−6 mol/L ghrelin, and washed three times with PBS. The cell layers were scraped into a solution containing 20 mM Tris–HCl, pH 8.0, and 150 mM NaCl, 1% Triton X-100, 0.02% NaN3 and 1 mM PMSF. After the lysates were homogenized by sonication for 20 s, the ALP activity was measured using an ALP kit.

### Measurement of mineralized matrix formation

For Alizarin Red S staining, CVSMCs in 24-well plates were cultured in medium that contained either 10−6 mol/L ghrelin or vehicle for 12 days. Then, the extent of mineralized matrix was determined by Alizarin Red S staining [34]. Briefly, cells were fixed in 70% ethanol for 1 h at room temperature and stained with 40 mM Alizarin Red S for 10 min. Next, cell preparations were washed with PBS to eliminate nonspecific staining.

For the quantification of calcium levels, cells were washed with PBS and decalcified with 0.6 N HCl for 24 h, Calcium content was determined by measuring the concentrations of calcium in the HCl supernatant by atomic absorption spectroscopy. After decalcification, the cells were washed three times with PBS and the cells were solubilized with 0.1 N NaOH/0.1% SDS. The protein content was measured with a BCA protein assay. The calcium content of the cell layer was normalized to the protein content.

### Silence of GHSR by RNA interference

RNA interference was used to silence the expression of GHSR in CVSMCs. GHSR-siRNA and scramble siRNA were synthesized by GenePharma Biotechnology (Shanghai, China). CVSMCs were plated in six-well plates and cultured for 24 h in medium without antibiotics. Cells were transfected with siRNAs (100 pmol/well) using Lipofectamine 2000 (Invitrogen) according to the manufacturer's instructions. Cells were cultured for 72 h. To detect calcium deposition 6 days after transfection, a second siRNA transfection was performed 72 h after the first time siRNA transfection as previously described [35], [36]. The efficiency of siRNA was determined by protein analysis.

### Western blot analysis

Total protein extracts of cultured cell were prepared with RIPA lysate (Beyotime, China) and equal amounts of protein were submitted to SDS-PAGE and transferred onto 0.2 µm PVDF membranes (Pall, USA) to be stained with appropriate antibodies (anti-p-p38, -p38, -p-ERK, -ERK, -p-JNK, -JNK, -Runx2, -GHSR and β-actin antibodies). The reaction was visualized with chemiluminescence.

### Real-time quantitative PCR assay for Runx2 and BMP mRNA expression

Total RNA was extracted by TRIzol (Invitrogen), and then cDNA was prepared with a RevertAid First Strand cDNA Synthesis Kit (Fermentas). The PCR was performed with Maxima SYBR Green/Rox qPCR Master Mix (Fermentas). All the procedures were strictly performed as per instructions. Amplification and detection were performed as follows: 50°C for 2 min, 95°C for 10 min and then 40 cycles of 95°C for 15 s, 60°C for 30 s, and 72°C for 30 s. A total 20 µl of reaction system consists of SYBR Mix 10 µl, Rnase-free water 7 µl, 1 µl/0.3 µmol/l forward/reverse primer and 1 µl/500 ng cDNA template. The results of real-time quantitative PCR were automatically analyzed by the Roche Light-Cycler technology. GAPDH was used as the inner control in this experiment. The primers used in this study were as follows: Runx2 sense: 5′-CCGGTCTCCTTCCAGGAT-3′, anti-sense: 5′-GGGAACTGCTGTGGCTTC-3′; BMP-2 sense: 5′-AGCTGCAAGAGACACCCTTT-3′, anti-sense: 5′-CATGCCTTAGGGATTTTGGA-3′; GAPDH sense: 5′-GGCTGCCCAGAACATCAT-3′, anti-sense: 5′-CGGACACATTGGGGGTAG-3′.

### Statistical analysis

Results were presented as means ± SD, and analysis was performed with Statistical Product and Service Solutions (version 13.0). Differences between groups were evaluated by one-way analysis of variance (ANOVA). The data shown were based on three independent experiments. A level of *p*<0.05 was considered significant.
